# Optoacoustic imaging in lower extremity revascularization: A novel technique to assess perioperative muscle perfusion

**DOI:** 10.1016/j.pacs.2025.100756

**Published:** 2025-07-31

**Authors:** Tim Wittig, Birte Winther, Charlene Reichl, Andrej Schmidt, Dierk Scheinert, Sabine Steiner

**Affiliations:** a Department of Angiology, University Hospital Leipzig, Leipzig 04103, Germany; b Helmholtz Institute for Metabolic, Obesity and Vascular Research (HI-MAG) of the Helmholtz Center Munich at the University of Leipzig and University Hospital Leipzig, Leipzig 04103, Germany; c iThera Medical GmbH, München 81379, Germany; d Division of Angiology, Department of Medicine II, Medical University Vienna, Vienna 1090, Austria

**Keywords:** Multispectral optoacoustic tomography, Optoacoustic, Muscle perfusion, Peripheral artery disease, Lower extremity revascularization

## Abstract

**Objectives:**

This proof-of-concept study aimed to assess the feasibility of Multispectral Optoacoustic Tomography (MSOT) in evaluating changes in oxygenated hemoglobin (HbO2) levels in muscles of the lower limb before and after lower extremity revascularization (LER).

**Methods:**

In 26 patients, HbO2 levels were assessed before and after LER, with follow-up assessing symptom control and patency for up to six months.

**Results:**

A significant difference in HbO2 levels was observed between pre- and post-LER in the muscles of the lower limb. In 10 patients, HbO2 levels did not increase following LER, and at the 6-month follow-up, 2 of these patients required target lesion revascularization (TLR) due to restenosis of ≥ 50 % stenosis. In contrast, 16 patients demonstrated increased HbO2 levels post-LER, with no patients requiring TLR at 6-months.

**Conclusion:**

This study demonstrates the potential of MSOT to detect changes in tissue perfusion following LER, highlighting its promise as a novel imaging modality for guiding treatment strategies.

## 1 Introduction

Peripheral arterial disease (PAD) affects over 230 million adults worldwide and is recognized as a significant contributor to global atherosclerotic cardiovascular morbidity [1]. Endovascular therapy (EVT) has become one of the preferred revascularization strategies to improve symptoms and achieve limb salvage, owing to its favorable acute outcomes and low periinterventional morbidity, especially in patients with high cardiovascular risk [2]. However, amputation rates remain high, partly due to the underuse of angiography and revascularization techniques, highlighting the importance of non-invasive diagnostic tools to guide early revascularization [3]. Current diagnostic approaches for PAD encompass clinical assessment, ankle-brachial index (ABI), and various imaging techniques, including duplex ultrasonography (DUS), magnetic resonance angiography (MRA), computed tomography angiography (CTA), and digital subtraction angiography (DSA), which primarily evaluate the macrovascular system. Currently, there is no method for real-time evaluation of microperfusion during treatment, highlighting a critical need for non-invasive diagnostic tools in the management of PAD [4].

Current techniques for measuring tissue perfusion include transcutaneous oxygen pressure (TcPO2), skin perfusion pressure (SPP) or near-infrared spectroscopy (NIRS). These modalities offer real-time measurements of perfusion in a clinical setting and have been incorporated into recent PAD guidelines [2]. However, TcPO2 and SPP provide insights at a few millimeters’ depth and NIRS allows measurement up to 1.5 cm, only representing perfusion in the epidermis and dermis [5], [6], [7]. Furthermore, these modalities have not yet been widely adopted in clinical practice due to a lack of standardization and low spatial resolution [2], [8].

Optoacoustic imaging (OAI) is an emerging technology that offers non-invasive, real-time visualization of endogenous absorbers, such as oxygenated and deoxygenated hemoglobin, in soft tissue. Using pulsed laser light in combination with ultrasound B-mode, this modality provides perfusion and oxygenation status in deeper tissue layers, including muscle tissue. With a penetration depth of up to 4 cm, Multispectral Optoacoustic Tomography (MSOT) has shown significant potential for clinical applications [9]. Over the past decade, MSOT has been utilized to quantify soft tissue composition in conditions such as cancer [10], [11], [12], inflammatory bowel disease [13], and neuromuscular disease [14], [15], [16]. In PAD patient, MSOT has already demonstrated its ability to correlate decrease in oxygenated hemoglobin with decrease in ABI [17], distinguish between healthy volunteers and patients with intermittent claudication [18], assess muscle degeneration [19], and quantify changes in perfusion after endovascular lower extremity revascularization (LER) [9].

In this proof-of-concept study, we aim to evaluate the feasibility of MSOT in assessing changes in lower limb tissue perfusion following EVT, using the tibialis anterior (TA) and flexor hallucis brevis (FHB) muscles as surrogates.

## 2 Methods

### 2.1 Study design and patient population

This prospective, proof-of-concept study was conducted in the department of angiology of the Leipzig University Hospital between January and August 2024. The study included patients presenting with moderate to severe intermittent claudication, ischemic rest pain and/or tissue loss (Rutherford category 2–5) who underwent EVT for subaortic lesions. A study team systematically documented patient data, procedural methodologies, and clinical outcomes through continuous patient monitoring. Ethics approval was obtained from the University of Leipzig Ethics Committee (approval no. 163/24-ek) and patients provided written informed consent before enrollment.

### 2.2 Study procedures

Patient characteristics and procedural data including lesion location, different types of balloons used (both uncoated and paclitaxel coated), use of adjunctive devices as well as information on concomitant in- and outflow interventions were collected through review of angiograms and clinical charts. As clinically indicated, ABI and DUS were performed during the pre-interventional evaluation and repeated before patient discharge. DSA was performed both before and after EVT. Patients received treatment in accordance with the standard protocol at our center independent of MSOT results. Technical success was defined as final in-lesion residual diameter stenosis of ≤ 50 % without device malfunction. Procedural success was defined as technical success without procedural complications [death, major target limb amputation, thrombosis of the target lesion, or target lesion revascularization (TLR)] prior to discharge.

**Fig. 1:**
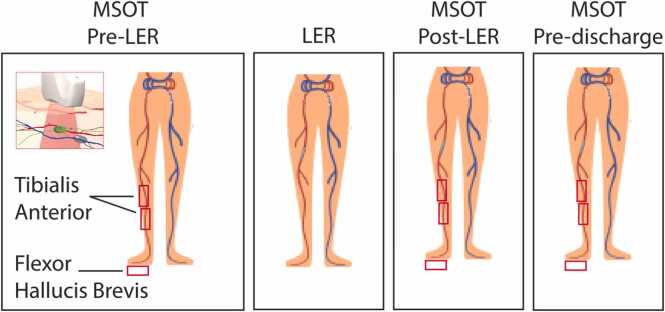
Positioning of the MSOT probe on the lower extremity. Indicated by the red rectangles are the following positions: tissue above the tibial anterior muscle proximal (TAP), tibial anterior muscle medial (TAM), and flexor hallucis brevis muscle (FHB).

Optoacoustic (OA) scanning was performed using the MSOT Acuity Echo CE system (Acuity©, iThera Medical GmbH, Munich, Germany) at predefined wavelengths. The ultrasound (US)/OAI system is equipped with a handheld probe with a center frequency of 4 MHz, a 256 elements transducer with an angular coverage of 125°, and a field of view of 40 × 40 mm (lateral and depth). The probe was used with an ultrasound probe cover and optimal coupling was ensured by using transparent ultrasound gel. MSOT was performed as follows: On the day of the intervention, patients were positioned on the procedure table and remained in a supine position for at least 10 min before the initiation of MSOT scanning. This rest period was implemented to ensure muscle relaxation and to minimize the influence of exercise- or movement-induced hyperemia, which could otherwise elevate perfusion signals. Scanning was conducted for all patients at baseline (prior to the intervention) and immediately following EVT, before the application of the pressure dressing. In a subset of patients (n = 18), a follow-up scan was conducted prior to discharge, within 24–48 h following the initial procedure. This scan was optional in the context of this proof-of-concept study and was limited by both the laser classification constraints and the fact that some patients had already been discharged. For the pre-discharge scan, patients were transported to the operating room, where the scanning protocol was performed as previously described, while patients remained in their beds. For scanning, the operator positioned the MSOT probe on each target muscle, including the TA (proximal [TAP] and medial [TAM] regions) and the FHB, as illustrated in Fig. 1. These positions were selected for optimal accessibility in the supine position, with the FHB included to evaluate foot perfusion. Each position was scanned three times. At baseline (pre-LER), before proceeding to the next scanning location, the position was marked on the patient’s skin using a surgical marker to facilitate precise repositioning during following assessments. US consistency assessment was performed during data analysis to evaluate the repositioning of the probe across timepoints. Since oxygenated hemoglobin (HbO2) has previously been shown to be a reliable biomarker for perfusion evaluation, we focused on this parameter [17].

### 2.3 MSOT data analysis

MSOT data analysis was conducted using iLabs software (version 1.3.33, iThera Medical GmbH). US consistency analysis was performed to evaluate the repositioning where three independent reviewers looked at the US pictures of TAP, TAM and FHB across all timepoints and scored them according to the similarity of the location between pre-LER, post-LER and pre-discharge. A score of 0 was given for US images showing differences across all timepoints, a score of 1 if two timepoints had the same location and a score of 2 if all three show reproducible positioning (Fig. S1). MSOT data included signals acquired at multiple wavelengths ranging from 700 to 870 nm (700, 710, 720, 730, 740, 750, 760, 770, 780, 790, 800, 810, 820, 830, 840, 850, 860, and 870 nm). Raw MSOT data were reconstructed using a back-projection algorithm, and the HbO2 signal was extracted via an unmixing algorithm based on linear regression using the absorption spectra of Hb and HbO2. Quantification was performed using iLabs software with semi-automated batch processing, following manual delineation of regions of interest (ROIs) on each scan. ROIs were positioned in the central region of the field of view (laterally) and in the upper portion of each muscle, avoiding blood vessels and image artifacts. In case of differences in US imaging between two timepoints, ROIs were adjusted along the lateral axis to maintain consistency. The mean signal within the ROI was calculated and expressed in arbitrary units. The raw data has been deposited in a data repository (https://opara.zih.tu-dresden.de/handle/123456789/1409).

### 2.4 Follow-up

Follow-up (FU) was conducted at 3 and 6 months, either through scheduled in-house visits with DUS or via telephone for patients undergoing outpatient ultrasound assessments. Clinical outcomes, Rutherford category, and the occurrence of safety events (death or amputation) were evaluated. Primary patency was defined as absence of TLR and/or binary restenosis (defined as a peak systolic velocity ratio > 2.4 as measured by US). TLR was defined as re-intervention for stenosis > 50 %. Good symptom control was defined as an improvement in Rutherford classification to stages 0–2. Patients were considered lost to FU if two unsuccessful contact attempts were made by phone and the primary physician could not be reached.

### 2.5 Statistical analysis

Continuous data are presented as mean ± standard deviation (SD), and normality was assessed using the Shapiro-Wilk test for all conditions (pre-, post-LER, and pre-discharge). A paired-samples *t*-test was performed to compare HbO2 signal changes between pre- and post-LER, as well as between pre-LER and pre-discharge, within the same group of patients. Pre-LER values were used as the baseline for comparison, enabling within-subject analysis. An increase in HbO2 value was considered when the normalized ratio was > 1.1, while values between 0 and ≤ 1.1 were interpreted as stable or decreased HbO2 values. Results are expressed as mean change ± SD. Pearson correlation coefficients were calculated to evaluate the linear relationship between HbO2 values post-LER and pre-discharge. Assumptions of linearity, normality, and the absence of significant outliers were verified before analysis. The correlation coefficient (r) and p-value are reported to describe the strength and direction of associations. All statistical analyses were performed using GraphPad Prism software (version 10.3.1 for Windows, GraphPad Software, Boston, Massachusetts USA, www.graphpad.com) and p-values < 0.05 were considered statistically significant.

## 3 Results

### 3.1 Study population and endovascular procedures

Between January and August 2024, a total of 28 patients with PAD scheduled for EVT were recruited from the Angiology Department at University Hospital Leipzig and received MSOT measurements peri-interventionally. Following data review, two patients were excluded from the analysis due to poor MSOT data quality, primarily caused by imaging artifacts such as prominent blood vessels or motion-related distortions. MSOT was performed at baseline and post-LER for all patients, with an additional scan conducted before discharge in a subset (n = 18). The mean age of the study population was 69.5 ± 7.2 years, with n = 21/26 (80.8 %) of participants identified as male and all patients were classified as light-skinned (n = 26/26, 100 %). Claudication was present in n = 18/26 (69.2 %) of patients, while n = 8/26 (30.8 %) presented with ischemic pain and/or ulceration. Cardiovascular risk factors were prevalent, with hypertension and hyperlipidemia observed in n = 25/26 (96.2 %) of patients and diabetes in n = 10/26 (38.5 %). Detailed patient characteristics are provided in Table 1. Procedural success and technical success were achieved in all patients (n = 26/26, 100 %). No patients required intra-procedural vasodilative medication during the intervention. Technical and procedural outcomes are summarized in Table 2. To correlate procedural details with MSOT findings, a procedural summary was documented for each patient.

**Table 1:** Baseline patient and lesion characteristics.

Characteristic	n = 26
**Age, yrs**	69.5 ± 7.2
**Male sex**	21 (80.8 %)
**History of**	
** Diabetes Mellitus Type 2**	10 (38.5 %)
** Hyperlipidemia**	25 (96.2 %)
** Arterial Hypertension**	25 (96.2 %)
** Coronary Heart Disease**	14 (53.9 %)
** Previous Stroke**	7 (26.9 %)
Renal Disease*	1 (3.9 %)
** Previous revascularization**	24 (92.3 %)
** Smoking**	
** never**	1 (3.8 %)
** current**	12 (46.2 %)
** former**	13 (50.0 %)
**Rutherford Classification**	
** 2**	2 (7.7 %)
**3**	16 (61.5 %)
**4**	3 (11.5 %)
**5**	5 (19.2 %)

Data are expressed as mean +/- SD, n (%) or median (interquartile range).

* Chronic kidney disease with a GFR < 30 mL/min/1.73 m².

**Table 2:** Lesion and procedural characteristics.

Characteristic	Optoacoustic Imaging (n = 26)
**Lesion type**	
**Aortoiliac**	2 (7.7 %)
**FemoroPopliteal**	21 (80.8 %)
**Infrapopliteal**	3 (11.5 %)
**Patent anterior tibial artery**	17 (65.4 %)
**Balloon angioplasty**	26 (100.0 %)
**Drug-coated balloon angioplasty**	22 (84.6 %)
**Stent implantation**	7 (26.9 %)
**Bare metal stent**	4 (15.4 %)
**Drug eluting stent**	1 (4.8 %)
**Directional atherectomy**	5 (19.2 %)
**Mechanical thrombectomy**	14 (53.8 %)
**Procedural success**	26 (100.0 %)
**Technical success**	26 (100.0 %)

Data are expressed as mean n (%).

### 3.2 Optoacoustic imaging results

**Fig. 2:**
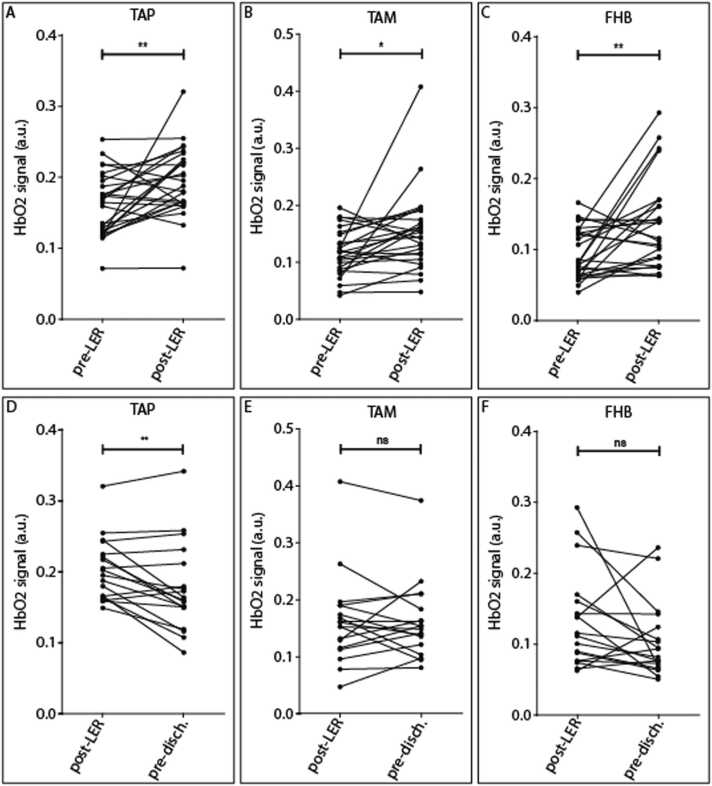
Change in oxygenated hemoglobin (HbO2) in the muscle of the lower extremity. Fig. 2A–D presents changes in HbO2 signals between pre- and post-LER over all regions for all patients (n = 26): A: Tibial anterior muscle proximal (TAP), B: Tibial anterior muscle medial (TAM) and C: Flexor hallucis brevis muscle (FHB). Fig. 2D–F presents changes in HbO2 signals between post-LER and pre-discharge in a subgroup of patients (n = 18) over all regions: D: Tibial anterior muscle proximal (TAP), E: Tibial anterior muscle medial (TAM) and F: Flexor hallucis brevis muscle (FHB).

**Fig. 3:**
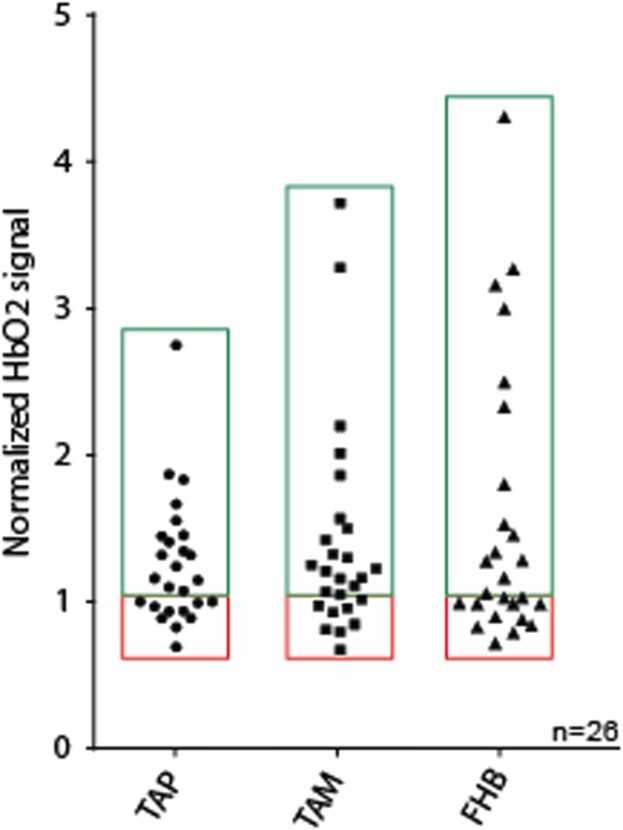
Normalized oxygenated hemoglobin (HbO2) values after lower extremity revascularization (post-LER). HbO2 signals in comparison to baseline before LER (pre-LER) in the tibial anterior muscle proximal (TAP), tibial anterior muscle medial (TAM), and flexor hallucis brevis muscle (FHB). Green and red rectangles symbolize increase or stable/decrease in HbO2 signal post-LER, respectively.

This study aimed to assess the feasibility of MSOT in evaluating changes in HbO2 levels in TAP, TAM, and FHB before and after EVT. Changes in MSOT signals are summarized in Table 3. Individual patient data, both raw and normalized, are presented in Fig. 2, Fig. 3, as well as in Table S1 and S2. The mean HbO2 levels were compared at baseline (pre-LER), post-LER and prior to discharge. As shown in Table 3 and Fig. 2 (A–C), there was a statistically significant difference in HbO2 signals between pre- and post-LER across all scanned areas, with p-values of < 0.01, 0.02, and < 0.01 for TAP, TAM, and FHB, respectively. Fig. 3 illustrates individual patients’ normalized HbO2 data (post-LER relative to pre-LER). HbO2 changes ranged from 0.69 to 2.75 in the TAP, 0.78 to 3.72 in the TAM, and 0.58 to 4.31 in the FHB and in 10 patients no improvement or even a decline in HbO2 signals could be identified post-LER.

**Table 3:** Comparison of pre- vs post-LER and post-LER vs pre-discharge: mean changes in optoacoustic signal.

**Scanned area**	**HbO2 pre-LER (a.u.)**	**HbO2 post-LER (a.u.)**	**Normalized change (to pre-LER)**	**p value**
**TAP**	0.162 (0.04)	0.194 (0.05)	1.267 (0.431)	0.0056 (**)
**TAM**	0.116 (0.04)	0.152 (0.07)	1.405 (0.720)	0.0157 (*)
**FHB**	0.094 (0.03)	0.137 (0.06)	1.63 (0.940)	0.0031 (**)
				
**Scanned area**	**HbO2 post-LER (a.u.)**	**HbO2 pre-discharge (a.u.)**	**Normalized change (to pre-LER)**	**p value**
**TAP**	0.203 (0.04)	0.177 (0.06)	1.122 (0.549)	0.0045 (**)
**TAM**	0.162 (0.08)	0.161 (0.07)	1.415 (0.662)	0.9862 (ns)
**FHB**	0.136 (0.07)	0.105 (0.05)	1.297 (0.807)	0.0573 (ns)

Data are expressed as mean (SD), p value was calculated using a paired *t*-test. n = 26 for pre- vs post-LER, n = 18 for post-LER vs pre-discharge.

* ‎p < 0.05.

** p < 0.01.

In addition, as shown in Table 3 and Fig. 2 (D–F), HbO2 signal increased significantly between post-LER and pre-discharge in the TAP, with a p-value of < 0.01. A strong positive correlation was observed between post-LER and pre-discharge HbO2 values (Pearson’s r = 0.83, n = 18, p < 0.01; Table S3, Fig. S2).

Among patients with decreased HbO2 signals after LER, n = 4/10 (40.0 %) had a patent anterior tibial artery (ATA) post-LER. In contrast, n = 13/16 patients (81.3 %) with increased HbO2 signals had a patent ATA post-LER. Among patients with a patent ATA (n = 17), we saw that even though slightly less significant in the TAP, all muscles showed a significant difference between pre- and post-LER (TAP p = 0.012, TAM p = 0.033, FHB p = 0.009) (Fig. S3).

Additionally, changes in deoxygenated hemoglobin (HbR) between pre- and post-LER (Fig. S4) show statistically significant differences in the TAM and FH but not in the TAP (p = 0.048, p = 0.008 and p = 0.061, respectively).

Finally, as shown in Table S4, patients suffering from IC (Rutherford 1–3) and CLTI (Rutherford 4–6) demonstrated an increase in HbO2 signal post-LER.

**Fig. 4:**
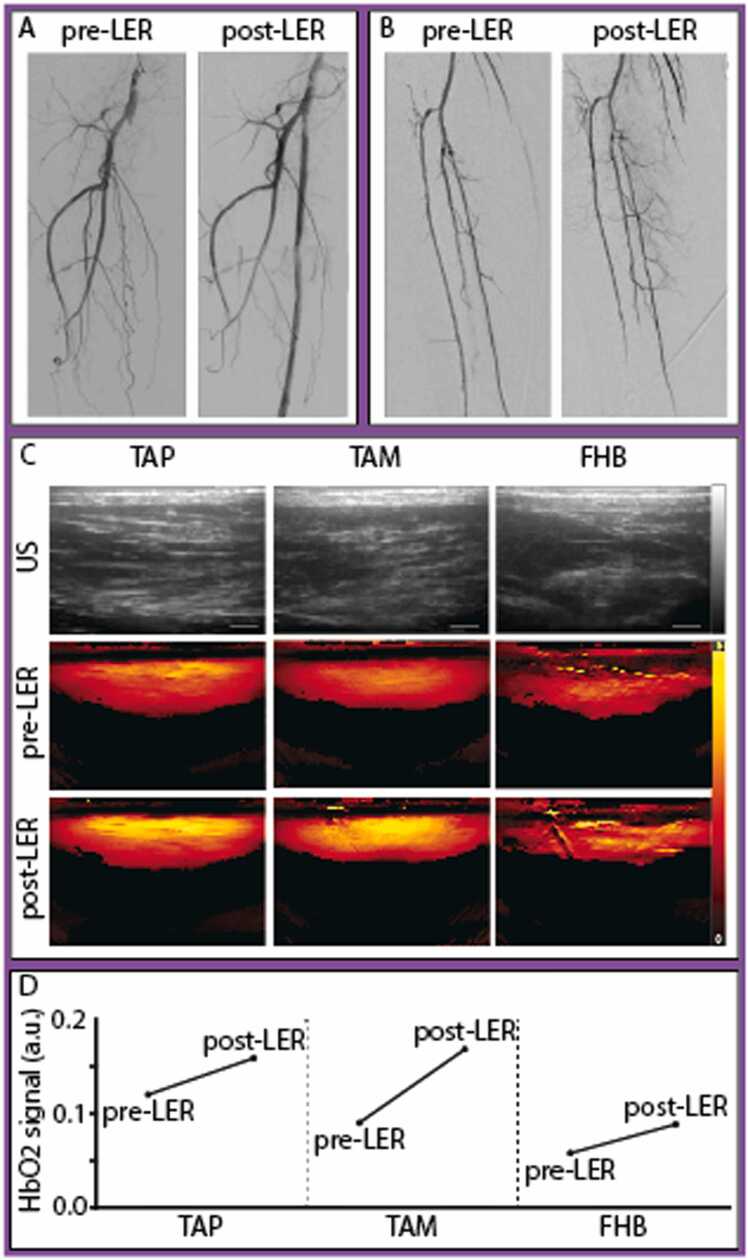
Angiography (A, B), optoacoustic (OA) (C), and oxygenated hemoglobin (HbO2) signals (D) of Patient 4 presenting with an in-stent occlusion of the right superficial femoral artery (SFA). Revascularization with rotational thrombectomy, balloon angioplasty, and stent implantation showed excellent results. After the procedure, the patient showed an increase in HbO2 signal in all muscles compared to baseline. At 6-month follow-up, the patient reported good patency and no claudication (Rutherford 0). A: Angiography of the SFA before and after lower extremity revascularization (LER). B: Angiography of the popliteal and infrapopliteal arteries after LER. C: US and OA images of the tibialis anterior proximal (TAP), tibialis anterior medial (TAM) and flexor hallucis brevis (FHB) pre- and post-LER with HbO2 signal legend from 0 (black) to 0.2 (yellow). D: Quantification of optoacoustic HbO2 signal pre- and post-LER in the tibialis anterior (proximal [TAP] and medial [TAM]) and flexor hallucis (FHB) after LER.

**Fig. 5:**
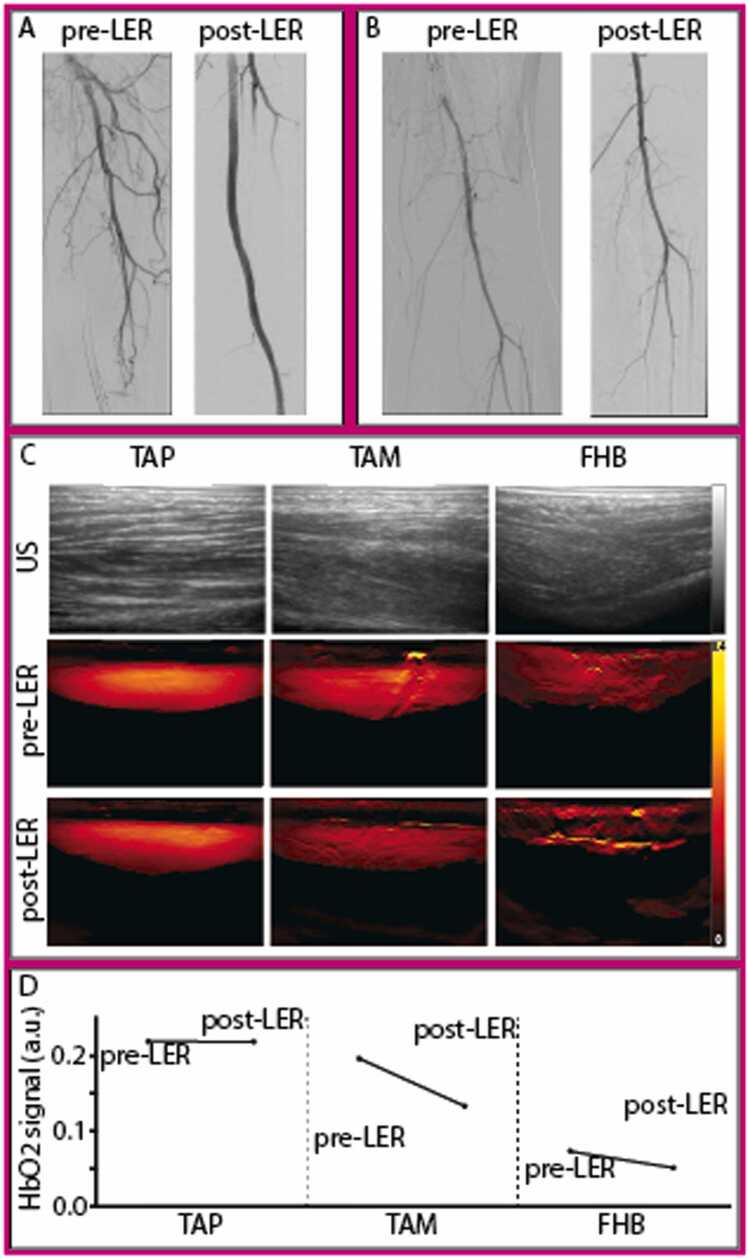
Angiography (A, B), optoacoustic images (C), and optoacoustic (OA) oxygenated hemoglobin (HbO2) signals (D) of Patient 3 presenting with an in-stent occlusion of the superficial femoral artery (SFA) who underwent rotational thrombectomy, balloon angioplasty, and stent implantation, achieving good angiographic results. After the procedure, the patient showed a decrease in HbO2 signal in all muscles compared to baseline. At the 3-month follow-up, the patient experienced target lesion revascularization (TLR) 66 days after the initial procedure. A: Angiography of the SFA before and after lower extremity revascularization (LER). B: Angiography of the popliteal and infrapopliteal arteries after LER. C: US and OA images of the tibialis anterior proximal (TAP), tibialis anterior medial (TAM) and flexor hallucis brevis (FHB) pre- and post-LER with HbO2 signal legend from 0 (black) to 0.4 (yellow). D: Quantification of optoacoustic HbO2 signal pre- and post-LER in the tibialis anterior (proximal TAP and medial TAM) and flexor hallucis (FHB) after LER.

Fig. 4 illustrates a case demonstrating a successful revascularization with corresponding MSOT values. This patient presented with an in-stent occlusion of the right superficial femoral artery (SFA). Following successful antegrade wire passage, revascularization was performed using rotational thrombectomy, drug-coated balloon angioplasty, and stent implantation. The procedure showed an excellent angiographic result, with no embolic events and three-vessel runoff. Correspondingly, MSOT revealed an increase in HbO2 signal across all scanned areas post-LER. At the 6-month FU, this patient demonstrated good primary patency and remained free of claudication (Rutherford 0). In contrast, Fig. 5 illustrates a case where successful revascularization was accompanied by decreased HbO2 signals in all scanned areas post-LER. This patient initially presented with an in-stent occlusion of the SFA and underwent treatment with rotational thrombectomy, (drug-eluting) balloon angioplasty, and stent implantation, achieving good angiographic results and a one-vessel run-off via the posterior tibial artery. However, at the 3-month follow-up, the patient experienced TLR 66 days after the initial procedure.

### 3.3 Follow-up

At the 6-month FU, data were available for 22 patients (n = 22/26, 84.6 %). Among the 10 patients who exhibited a decrease in HbO2 signal following LER, 1 patient (10.0 %) had died from non-procedure-related causes 201 days after the procedure and 1 (10.0 %) was lost to follow-up. Two patients (20.0 %) experienced TLR, with both patients requiring reintervention within 3 months. Six patients (60.0 %) reported good primary patency and symptom control (Rutherford 0–2).

Among the 16 patients with improved HbO2 signals post-LER, 2 (12.5 %) were lost to follow-up, 13 (81.3 %) reported good primary patency and symptom control (Rutherford 0–1), and 1 patient (6.2 %) with an ulceration of the affected limb was still in the healing process (Rutherford 5). No patients required minor or major limb amputation.

## 4 Discussion

Current clinical practice lacks a reliable tool for real-time assessment of tissue perfusion, during and after EVT. Previous research has demonstrated the sensitivity of MSOT in detecting muscle degeneration, an early indicator of PAD, resulting from chronic tissue mal-perfusion [19], [20]. We aimed to assess the feasibility of MSOT in monitoring lower limb tissue perfusion before and after LER in PAD patients, using the TA and FHB muscles as surrogates.

In this proof-of-concept study, MSOT showed a possible potential to depict changes in tissue perfusion following EVT. A previous study demonstrated the ability of MSOT to detect changes in tissue perfusion in the muscle of healthy volunteers undergoing cuff-induced arterial occlusion [9]. Additionally, MSOT successfully quantified tissue perfusion changes in the calf muscles before and 24 h after revascularization in patients undergoing balloon and stent angioplasty of the SFA and popliteal artery, showing a 1.6-fold increase in HbO2 [9]. In our study, we observed up to 4-fold increase in HbO2 immediately after LER over all regions, further supporting MSOT as a potential non-invasive tool for image-guided treatment. Moreover, a strong positive correlation was observed between post-LER and pre-discharge HbO2 values, indicating that changes in HbO2 signals immediately after LER generally persisted until discharge. This suggests that a significant hyperemic muscle response following reperfusion was not present at the time of scanning, reinforcing the potential of MSOT as a reliable tool for intra- and perioperative evaluation of muscle perfusion. Furthermore, the observed changes in HbR indicate that this parameter should be evaluated alongside HbO2. Considering both parameters might allow for a more comprehensive assessment of muscle perfusion and function following revascularization.

Among 10 patients with stable or decreased HbO2 levels after LER, 2 required TLR within 3 months. In contrast, none of the patients who showed increased HbO2 signals after LER underwent TLR at 6 months. Notably, not all patients with decreased or stable HbO2 levels after LER experienced TLR and most reported good symptom control at 6-months. Since all measurement areas were perfused by the ATA, untreated ATA stenosis or occlusion may account for the lack of improvement in HbO2 levels. Among the patients who experienced a decrease in HbO2 signal after LER, 40.0 % had a patent ATA post-LER. In contrast, among those with increased HbO2 signals, 81.3 % had a patent ATA post-LER. Given the variability in patient outcomes further investigation is warranted to determine whether ATA patency is a critical is essential for predicting perfusion in these regions. Further studies utilizing alternative measurement sites will also be necessary to validate this hypothesis. Additionally, slow-flow phenomena or micro-embolisms may have contributed to the absence of an HbO2 increase. Initial slow-flow phenomena have been previously reported with a prevalence of up to 18 % following PTA [21]. Micro-embolisms in the microcirculatory system have been associated with plaque excision and stent implantation, typically without significant clinical consequences [22]. This could further explain the absence of an increase in HbO2 levels despite the lack of clinical sequelae.

Further studies are needed to evaluate the clinical applications of MSOT; however, initial results are promising. MSOT has already demonstrated its ability to differentiate between healthy volunteers and PAD patients [9]. Additionally, MSOT has shown potential in predicting PAD severity and detecting critical perfusion deficits, even in cases where standard diagnostic methods were inconclusive [17]. Future research could investigate the potential role of this method in intraoperative, image-guided decision-making. Effective perfusion assessment during LER may facilitate a quantitative evaluation of restored blood flow in the lower limb, providing real-time feedback on whether significant hemodynamic improvements have been achieved. This could be especially important for patients with ulceration, as it may help ensure adequate perfusion for wound healing. Furthermore, hemodynamic assessment may aid in quantifying the severity of vessel dissection and provide valuable information when determining the need for bail-out stenting.

However, at this stage, technical limitations of MSOT require further optimization to advance clinical implementation. First, software improvements enabling precise repositioning pre- and post-intervention would enhance longitudinal scan consistency, particularly for follow-up assessments. Second, downgrading the laser classification from Class 4–3B would simplify safety protocols for both operators and patients. At the time of the study, the laser system remained classified as Class 4, requiring operation within a radiation-controlled environment such as a catheterization laboratory. This imposed logistical challenges for follow-up examinations. In addition, the use of laser safety goggles was mandatory to ensure operator and patient protection. Lastly, reducing the number of wavelengths—or adopting single/dual-wavelength acquisition—would shorten scan time, mitigating motion artifacts. Eliminating spectral unmixing could further reduce the risk of spectral coloring. Once refined, this technique holds the potential to reduce short-term readmissions, shorten procedural duration, minimize exposure to ionizing radiation and contrast agents, and lower the risk of complications.

The primary limitation of this pilot study is the small sample size, along with the absence of a control group to validate our findings. Additionally, motion or other image artefacts required the exclusion of 2 patients, which could be solved in future studies by reducing the number of wavelengths used during acquisition. Furthermore, assessing direct muscle perfusion would yield a more precise evaluation of tissue-level ischemia and offer critical insight into the effectiveness of revascularization. Lastly, the penetration depth of the OAI modality may be limited in patients with increased subcutaneous tissue or edema. This constraint has also been observed during the analysis of muscle perfusion in the optoacoustic signal. Future advancements, such as the implementation of reliable fluence correction algorithms, may help to overcome this limitation.

## 5 Conclusion

This proof-of-concept study demonstrates MSOT potential to possibly depict changes in tissue perfusion following EVT, using muscles of the lower limb as surrogates. These findings suggest that MSOT holds promising potential as a novel imaging modality to guide treatment and enhance outcomes following EVT in patients suffering from PAD.

## CRediT authorship contribution statement

**Sabine Steiner:** Writing – review & editing, Validation, Supervision, Project administration, Conceptualization. **Birte Winther:** Writing – original draft, Visualization, Resources, Investigation, Conceptualization. **Charlene Reichl:** Writing – original draft, Visualization, Software, Methodology, Formal analysis, Data curation, Conceptualization. **Andrej Schmidt:** Writing – review & editing, Supervision, Methodology, Investigation, Conceptualization. **Dierk Scheinert:** Writing – review & editing, Supervision, Conceptualization. **Tim Wittig:** Writing – original draft, Visualization, Validation, Resources, Methodology, Investigation, Data curation, Conceptualization.

## Funding

No funding.

## Declaration of Competing Interest

The authors declare the following financial interests/personal relationships which may be considered as potential competing interests: Charlene Reichl reports a relationship with IThera Medical GmbH that includes: employment. Andrej Schmidt reports a relationship with Abbott Laboratories Inc that includes: consulting or advisory. Andrej Schmidt reports a relationship with Becton Dickinson and Company that includes: consulting or advisory. Andrej Schmidt reports a relationship with Boston Scientific Corporation that includes: consulting or advisory. Andrej Schmidt reports a relationship with Cook Medical Inc that includes: consulting or advisory. Andrej Schmidt reports a relationship with Reflow Medical, Inc. that includes: consulting or advisory. Andrej Schmidt reports a relationship with Upstream Peripheral Technologies that includes: consulting or advisory. Dierk Scheinert reports a relationship with Abbott Laboratories Inc that includes: consulting or advisory. Dierk Scheinert reports a relationship with Acotec that includes: consulting or advisory. Dierk Scheinert reports a relationship with Boston Scientific Corporation that includes: consulting or advisory. Dierk Scheinert reports a relationship with Concept Medical Inc. that includes: consulting or advisory. Dierk Scheinert reports a relationship with Medtronic that includes: consulting or advisory. Dierk Scheinert reports a relationship with Upstream Peripheral Technologies that includes: consulting or advisory. Dierk Scheinert reports a relationship with Penumbra Inc that includes: consulting or advisory. Dierk Scheinert reports a relationship with Philips that includes: consulting or advisory. Dierk Scheinert reports a relationship with Reflow Medical, Inc. that includes: consulting or advisory. Sabine Steiner reports a relationship with AngioDynamics Inc that includes: consulting or advisory. Sabine Steiner reports a relationship with BIOTRONIK Inc that includes: consulting or advisory. Sabine Steiner reports a relationship with Boston Scientific Corporation that includes: consulting or advisory. Sabine Steiner reports a relationship with Cook Medical Inc that includes: consulting or advisory. Sabine Steiner reports a relationship with IThera Medical GmbH that includes: consulting or advisory. If there are other authors, they declare that they have no known competing financial interests or personal relationships that could have appeared to influence the work reported in this paper.

## Data Availability

The raw data has been deposited in a data repository.

## Glossary

ABI: Ankle-brachial index
AHA: American Heart Association
ATA: Anterior tibial artery
CTA: Computed tomography angiography
DUS: Duplex ultrasonography
EVT: Endovascular therapy
FHB: Flexor hallucis brevis muscle
FU: Follow-up
HbO2: Oxygenated hemoglobin
LER: Lower extremity revascularization
MRA: Magnetic resonance angiography
MSOT: Multispectral optoacoustic tomography
NIRS: Near-infrared spectroscopy
OA: Optoacoustic
OAI: Optoacoustic imaging
PAD: Peripheral arterial disease
ROI: Regions of interest
SD: Standard deviation
SFA: Superficial femoral artery
SPP: Skin perfusion pressure
TAM: Tibialis anterior muscle, medial
TAP: Tibialis anterior muscle, proximal
TcPO2: Transcutaneous oxygen pressure
TLR: Target lesion revascularization
US: Ultrasound
