# Supplementary material for: Optoacoustic imaging in lower extremity revascularization: A novel technique to assess perioperative muscle perfusion
Source: Photoacoustics. 2025 Jul 31;45:100756. doi: 10.1016/j.pacs.2025.100756 (PMC12337640; doi:10.1016/j.pacs.2025.100756)
Supplement: Supplementary file 1 — Supplementary material [file mmc1.docx]

**SUPPLEMENTARY MATERIAL**

Table S1. Change in MSOT signal between post and pre-LER correlated with technical and procedural success for all patients. Patients with stable/decreased MSOT signal marked in red.

| Patient # | Technical Success | Procedural Success | Change in HbO2 (normalized to pre-LER) | | |
| --- | --- | --- | --- | --- | --- |
|  |  |  | TAP | TAM | FHB |
| 1 | Yes | Yes | 0.892 | 0.852 | 1.290 |
| 2 | Yes | Yes | 1.874 | 3.283 | 0.902 |
| 3 | Yes | Yes | 0.997 | 0.676 | 1.025 |
| 4 | Yes | Yes | 1.329 | 1.880 | 1.531 |
| 5 | Yes | Yes | 1.413 | 0.799 | 1.167 |
| 6 | Yes | Yes | 1.670 | 1.112 | 0.989 |
| 7 | Yes | Yes | 1.164 | 1.019 | 3.163 |
| 8 | Yes | Yes | 0.971 | 1.306 | 0.793 |
| 9 | Yes | Yes | 1.006 | 1.162 | 0.833 |
| 10 | Yes | Yes | 1.350 | 1.571 | 2.502 |
| 11 | Yes | Yes | 0.696 | 1.425 | 3.001 |
| 12 | Yes | Yes | 1.152 | 0.815 | 3.272 |
| 13 | Yes | Yes | 0.831 | 1.055 | 0.843 |
| 14 | Yes | Yes | 1.559 | 2.203 | 0.996 |
| 15 | Yes | Yes | 1.837 | 2.016 | 0.991 |
| 16 | Yes | Yes | 1.324 | 1.075 | 0.884 |
| 17 | Yes | Yes | 1.081 | 0.964 | 1.503 |
| 18 | Yes | Yes | 0.940 | 0.979 | 1.397 |
| 19 | Yes | Yes | 1.246 | 1.168 | 1.229 |
| 20 | Yes | Yes | 0.939 | 0.930 | 1.771 |
| 21 | Yes | Yes | 0.893 | 1.215 | 1.110 |
| 22 | Yes | Yes | 1.459 | 1.330 | 2.293 |
| 23 | Yes | Yes | 1.453 | 1.504 | 1.477 |
| 24 | Yes | Yes | 1.006 | 1.255 | 4.223 |
| 25 | Yes | Yes | 1.103 | 1.229 | 2.532 |
| 26 | Yes | Yes | 2.719 | 3.721 | 0.673 |

Table S2. Change in MSOT signal between pre-discharge and pre-LER and pre-discharge and post-LER for all patients.

| Patient # | Change in HbO2 (normalized to pre-LER) | | | | | Change in HbO2 (normalized to post-LER) | | | |
| --- | --- | --- | --- | --- | --- | --- | --- | --- | --- |
|  | TAP | TAM | | FHB | | TAP | | TAM | FHB |
| 1 | 0.576 | 0.526 | | 1.813 | | 0.475 | | 1.475 | 2.424 |
| 2 | 1.928 | 3.016 | | 1.101 | | 0.321 | | 0.532 | 1.710 |
| 3 | 0.725 | 0.839 | | 0.707 | | 0.266 | | 1.205 | 2.270 |
| 4 | 1.261 | 1.155 | | 1.108 | | 0.776 | | 0.989 | 1.081 |
| 5 | 0.736 | 1.011 | | 1.161 | | 0.386 | | 2.310 | 0.801 |
| 6 | 1.129 | 1.487 | | 1.948 | | 0.369 | | 1.781 | 0.738 |
| 7 | 1.201 | 2.115 | | 1.792 | | 0.713 | | 3.414 | 0.591 |
| 8 | 1.051 | 1.274 | | 0.710 | | 0.474 | | 0.473 | 0.580 |
| 17 | 0.856 | 1.207 | | 1.002 | | 0.319 | | 1.448 | 1.081 |
| 18 | 0.621 | 0.762 | | 1.026 | | 0.383 | | 1.397 | 2.104 |
| 19 | 1.300 | 0.947 | | 1.179 | | 0.325 | | 0.873 | 1.014 |
| 20 | 1.017 | 0.962 | | 0.699 | | 0.360 | | 2.506 | 0.922 |
| 21 | 0.805 | 2.190 | | 1.298 | | 0.655 | | 1.455 | 2.967 |
| 22 | 1.180 | 1.425 | | 0.546 | | 0.615 | | 1.247 | 0.553 |
| 23 | 0.973 | 1.506 | | 0.827 | | 0.232 | | 1.573 | 1.117 |
| 24 | 1.019 | 1.392 | | 3.895 | | 0.157 | | 0.784 | 0.668 |
| 25 | 0.882 | 1.062 | | 2.061 | | 1.116 | | 1.977 | 2.601 |
| 26 | 2.934 | 2.596 | 0.465 | 0.551 | 0.061 | | 0.426 | | |

Table S3. Change in HbO2 signal between pre- and post-LER based on Rutherford classification.

| Scanned area | Normalized change (to pre-LER) | |
| --- | --- | --- |
|  | **Rutherford 1-3** | **Rutherford 4-5** |
| TAP | 1.347 (0.457) | 1.085 (0.321) |
| TAM | 1.527 (0.836) | 1.130 (0.185) |
| FHB | 1.504 (0.715) | 1.912 (1.333) |

Table S4. HbO2 signal correlation matrix between pre-LER, post-LER and pre-discharge

|  | *TAP HbO2 pre-LER* | *TAP HbO2 post-LER* | *TAP HbO2 pre-discharge* |
| --- | --- | --- | --- |
| TAP HbO2 pre-LER | 1 |  |  |
| TAP HbO2 post-LER | 0,2957857 | 1 |  |
| TAP HbO2 pre-discharge | 0,09563789 | 0,8311776 | 1 |

Figure S1. Ultrasound consistency rating. A score of 0 was given for US images showing differences across all timepoints, a score of 1 if two timepoints have the same location and a score of 2 if all three show reproducible positioning

Figure S2. Scatter plot of HbO2 signal post-LER and pre-discharge for all muscles.

Figure S3. Change in oxygenated hemoglobin (HbO2) in the muscle of the lower extremity in patients with patent ATA in the Tibial anterior muscle (TAP) proximal, Tibial anterior muscle medial (TAM) and Flexor hallucis brevis muscle (FHB) between pre-LER and post-LER.

Figure S4. Change in deoxygenated hemoglobin (HbR) in the muscle of the lower extremity in the Tibial anterior muscle (TAP) proximal, Tibial anterior muscle medial (TAM) and Flexor hallucis brevis muscle (FHB) between pre-LER and post-LER.
